# Comparative Analysis of Load Profile between Small-Sided Games and Official Matches in Youth Soccer Players

**DOI:** 10.3390/sports6040173

**Published:** 2018-12-12

**Authors:** Carlos David Gómez-Carmona, José Martín Gamonales, José Pino-Ortega, Sergio José Ibáñez

**Affiliations:** 1 Optimization of Training and Sports Performance Research Group (GOERD), Department of Didactics of Music, Plastic and Body Expression, Sports Science Faculty, University of Extremadura, 10004 Caceres, Spain; martingamonales@unex.es (J.M.G.); sibanez@unex.es (S.J.I.); 2 Department of Physical Activity and Sport Science, Sport Science Faculty, University of Murcia, 30720 San Javier, Spain; josepinoortega@um.es

**Keywords:** team sports, game-based training, external load, internal load, inertial devices

## Abstract

The purposes of the present study are: (a) to characterize the external (eTL) and internal load (iTL) of official matches and small-sided games (SSGs) in relation to their objective, (b) to compare demands between SSG, and (c) to analyze the SSG requirements in relation to official matches during a one-month competition period. Twenty under-18 national-level soccer players were recorded using WIMU^TM^ inertial devices (RealTrack Systems, Almeria, Spain) during four official matches and 12 training sessions where four SSGs with different objectives were performed: (SSG1) keeping the ball; (SSG2) keeping the ball and progressing; (SSG3) keeping the ball, progressing and ending in mini-goals; and (SSG4) keeping the ball, progressing and ending in an official goal with a goalkeeper. Statistical analysis included Kruskall-Wallis’ H and Mann-Whitney’s U with Cohen’s d effect size. The SSGs presented walking and jogging intensity movements (0.7–7 to 7–14 km/h), with a 5-to-8 %HIA (high intensity activity, >16 km/h), where low intensity accelerations, decelerations and impacts were predominant (1–2.5 m/s^2^; 5–7 G), and %HR_MAX_ (maximum heart rate percentage) was between 70–90%. Only SSG4 presented similar demands to competition, finding differences between SSGs (*p* < 0.05; *d* = 1.40 − 0.36). In conclusion, the objective of the SSGs directly influenced the demands on the players in training sessions. For this reason, it is important to monitor demands for designing specific training sessions.

## 1. Introduction

Soccer is a team sport with high-intensity intermittent actions, where performance depends on different technical, tactical, biomechanical, psychological and physiological aspects [1]. Currently, training methodology is one of the most studied aspects [2,3] with the aim of designing more specific training sessions, where small-sided games (SSGs) are one of the most commonly used tasks [4,5]. SSGs are training tasks with a similar structure to the real game, that are performed in small areas of the pitch with adapted rules and a reduced number of players compared to official games [6,7]. Besides, these situations reproduce similar aerobic demands to competition [8].

To design the SSGs, a number of variables have been studied such as: (a) the players’ area [9], (b) the size of the pitch [8,10], (c) the field orientation [11,12], (d) the number of players [5,13], (e) the type of defense, (man-to-man or zone) [14], (f) the work-rest ratio [10,15], (g) the specific objectives, ending or ball possession [16,17], (h) the modification of the game rules [18], (i) play with or without a goalkeeper [19], (j) the presence of wildcard players [20] and (k) the feedback of the coach [8,21], among others. All of these are modified with the aim of reproducing the specific demands of the game [10], as they are effective independently of age, sex, experience and competitive level [22,23].

Thanks to the technological advances of the last several years, new inertial measurement units (IMUs) composed of many sensors for time-motion analysis (accelerometers, gyroscopes or magnetometers) and tracking location in indoor (Local Position Measurement, LPM; Ultra-Wide Band, UWB) [24,25] and outdoor conditions (Global Navigation Satellite Systems, GNSS) [26,27] have been developed to analyze movements, velocity, distance covered, number of accelerations and decelerations [28,29,30], to know the specific requirements of soccer [31]. Different publications have analyzed the internal load imposed by soccer through objective methods such as heart rate (HR) and subjective methods such as rating perceived exertion (RPE) [10,32,33].

SSGs do not exactly reproduce the real game context [10,33,34]. In addition, these game-based tasks can exceed the ratio of changes of speed and could increase injury risk [7]. For this reason, it is hypothesized that the demands of the SSGs are not related to competition demands and the modification of the objective in game-based tasks will have an influence on their requirements. Therefore, this investigation was proposed due to the lack of research in relation to the comparison between SSGs and official match demands and the analysis of requirements in youth soccer players. The aims of the present study are: (a) to characterize the external (eTL) and internal load (iTL) of official matches and small-sided games in relation to their objective, (b) to compare demands between SSGs, and (c) to analyze SSG requirements in relation to official match load during a one-month competition period in youth national-level soccer players.

## 2. Materials and Methods

### 2.1. Design

The research is based on a cross-sectional study with natural groups [35]. The investigation characterizes the external and internal load of official matches and small-sided games and compares their demands in youth soccer players during a one-month competition period.

### 2.2. Participants

Twenty under-18 national-level soccer players (age: 17.32 ± 0.87 years; body mass: 67.27 ± 5.78 kg; height: 1.72 ± 0.08 m; Body Mass Index (BMI): 21.83 ± 1.89 kg/m^2^) who play in a semi-professional club participated voluntarily in the research. The participants in the present study presented the following inclusion criteria: (1) no physical limitations or musculoskeletal injuries that could affect testing, (2) participating up to 70-min of total duration in official matches, and (3) goalkeepers were not included in the study because their physical load differs from all other field players. Finally, only 16 players were analyzed, two goalkeepers and two players being excluded. Participants were informed about the research procedures and provided written informed consent. The testing protocol was approved by the University ethics committee (register number 67/2017).

### 2.3. Sample

A total of 229 statistical analysis units were recorded during the research, composed of 37 recordings in competition (10 field players in four official matches, without the goalkeeper) and 192 recordings during training sessions (four SSGs repeated in four training sessions, one per week, performed by 12 players that were distributed in two teams), only being recorded the day that SSGs were performed.

### 2.4. Equipment

WIMU^TM^ inertial devices (RealTrack Systems, Almeria, Spain) were utilized for data acquisition. This device is composed of different sensors (four accelerometers with different full scale: ± 16 g, ± 16 g, ± 32 g and ± 400 g; a gyroscope with a full scale of 2000 degrees/second; a magnetometer, GPS chipset, among others) for movement analysis and to track location in outdoor conditions [27] showing a high degree of accuracy (≈ 50 cm) [24]. External load demands were recorded: (a) tracking data by GPS with a sample frequency of 10 Hz, and (b) movement data through the IMU accelerometers with a sample frequency of 100 Hz. Heart rate (HR) was utilized to record internal load demands with a GARMIN^TM^ HR band (Garmin Ltd., Olathe, KS, USA), that sent data to the inertial device through Ant+ technology [36]. During the present study, all data were recorded in the 8 GB internal memory of each device. During the session, the selections of activity time in each small-sided game or match halves were carried out in real-time by SVIVO^TM^ software (version 807, RealTrack Systems, Almeria, Spain). Finally, all devices and the activity marks were imported in a computer and analyzed by SPRO^TM^ (RealTrack Systems, Almeria, Spain).

### 2.5. Variables

The present study analyzed the following independent and dependent variables shown in Table 1 and Table 2. The external load variables were divided into two groups: (a) kinematical and (b) neuromuscular [37]. The kinematical variables analyzed were: (i) total relative distance covered and at different speeds, (ii) high-intensity activity (HIA), (iii) speed average, (iv) accelerations, (v) decelerations, and (vi) metabolic power. The speed thresholds for relative distance and high-intensity activity are similar to those employed in previous time–motion studies [38,39,40]. The intensity of accelerations and decelerations were classified according to [41]. Metabolic power was calculated following [42]. In relation to neuromuscular load, the recorded variables were PlayerLoad^TM^ and impacts at different ranges. PlayerLoad^TM^ was calculated following [43] and impacts were classified according to [44]. Finally, to quantify internal load, heart rate (HR) variable at all intensities was recorded. Maximum HR (HR_MAX_) of each player was selected individually through the highest value recorded in official matches, the different intensities being classified according to [45].

### 2.6. Procedures

The present study was performed during the competitive period of the 2017–2018 season, between January and February 2018. The one-month competitive period was composed of three training sessions that were classified in relation to Match Day (MD) (Monday: MD + 2, Wednesday: MD − 3, and Friday: MD − 1) and an official match per week. The training sessions always started at 21:00 h and had a total average duration of 114 ± 7 min. The SSGs were played at MD − 3 on an artificial grass pitch (regular field surface) with dimensions of 25 m length and 40 m width, related to the short narrow pitch described by Casamichana et al. (2018) [46]. These dimensions were selected as it is a common game-based task used in soccer called “double-area” and classified by Rampinini et al. (2007) as small field dimensions [8]. Each SSG was performed twice, where ball possession was exclusively for one team, thus, if the defending team stole the ball, it was given back to the attacking team. Besides, additional balls were distributed around the edge of the pitch to maximize effective playing time, and were passed to the attacking team [47]. In SSG1, the ball was passed to any attacking player, while, in SSG2, SSG3 and SSG4 the ball was passed to the attacking player who was nearest to the attacking start line. The duration of each repetition was 5 min with a 2-min rest interval between SSGs. The participants were distributed in two teams of six players, without specific roles, except in SSG4 where seven players took part in the defending team (6 players plus a goalkeeper). During the SSGs, the coach did not give feedback to the players. Regarding the official matches, these lasted 90 min (two halves of 45 min) and they were played on the same surface as the SSGs so as not to contaminate the research.

To place the devices on the players, they were cited 15-min before the training sessions and 60-min before the official matches. Firstly, before placing the device on each athlete, it was calibrated through an internal process during auto-starting following the manufacturer’s recommendations: (i) to switch on the devices in a flat zone and without contact with other magnetic devices, (ii) and for them to remain still for approximately 30 s after switching them on, (iii) once the operating system of the device was initialized, a button was pressed in order to start recording. Then, the devices were placed on every player using a specific anatomical harness.

### 2.7. Statistical Analysis

Firstly, a descriptive analysis was performed showing the data as mean and standard deviation (mean ± SD) to describe the internal and external load demands of small-sided games, in relation to their aims, and the official matches analyzed during a four-week period. Then, an exploratory analysis was done for criteria assumption [48]. Table 3 shows the results of the *Kolmogorov-Smirnov* test for *Normality* and the *Levene* test for *Homoscedasticity* that were performed to establish the correct model for hypothesis contrast 3.

Finally, an inferential analysis was done to compare the internal and external load demands between official matches and small-sided games designed in relation to the objective. *Kruskall-Wallis’ H* and *Mann-Whitney’s U* for pairwise comparison were used. Significance level was established at *p* < 0.05 [48]. Additionally, Cohen’s d was calculated to obtain the magnitude of differences through the effect size calculator for non-parametric tests (www.psychometrica.de/effect_size.html), classifying them as very low (0–0.2), low (0.2–0.6), moderate (0.6–1.2), high (1.2–2.0) and very high (>2.0) [49]. The Statistical Package of Social Science (version 24.0; SPSS Inc., Chicago, IL, USA) was used for the statistical analysis and GraphPad Prism (version 7; GraphPad Software, La Jolla, CA, USA) to design the plots.

## 3. Results

Table 4 shows the descriptive analysis of external and internal load variables recorded in the investigation in relation to the small-sided games and official matches. The small-sided games demand profile is characterized by movements performed at walking and jogging intensity (0.7–7 km/h and 7–14 km/h), at 5-to-7 km/h average speed, with a 5-to-8 %HIA, and where the low intensity accelerations, decelerations and impacts were predominant (1–2.5 m/s^2^; 5–7 G). With respect to internal load, the average heart rate was 155-to-167 bpm, for this reason, the percentage of HR_MAX_ was between 70–90%.

In the comparison analysis in relation to the objective of the SSGs, statistical differences were found in all the variables recorded (*p* < 0.05; *d* = 1.40 − 0.36) with a moderate-to-high effect size, except in [8–10 G] Imp/min, [>10 G] Imp/min, [50–60%] HR_MAX_, and [95–100%] HR_MAX_. SSG1 and SSG4 presented the highest demands in external and internal training load variables; while SSG3 obtained the lowest demands.

**Figure 1:**
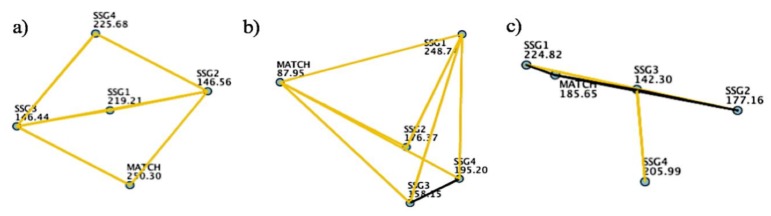
Pairwise comparison in (**a**) %HIA, (**b**) Acc/min, and (**c**) HR_AVG_ in relation to small-sided games and official matches analyzed. The yellow line represents significant differences among groups (*p* < 0.05), while the black line represents non-significant differences among groups (*p* > 0.05).

**Figure 2:**
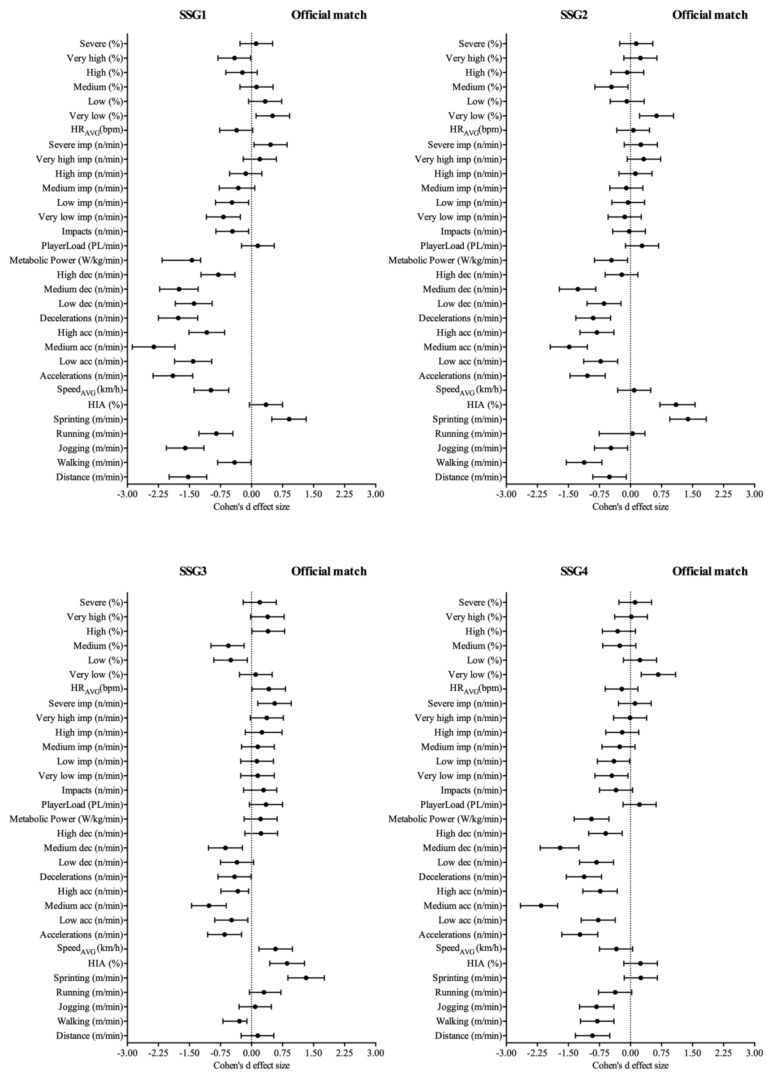
Dot plot to represent the pairwise comparison between SSG and official match demands in the external and internal load variables analyzed in the present research by Cohen’s d effect size with 95% CI.

Finally, in relation to match demands profile, the U-18 soccer players covered 98.30 ± 21.94 m/min at an average speed of 5.95 ± 0.94 and with a 9.74 ± 4.21% of HIA. Besides, the players performed 12.42 ± 2.28 Acc/min, 10.85 ± 2.26 Dec/min, 6.24 ± 1.31 W/kg/min, 1.80 ± 2.95 PL/min and 10.46 ± 6.62 Imp/min, with an average HR of 161 ± 15 bpm. With respect to the official match demands mentioned before, the SSG depending on the objective presented a different load profile, as shown in Figure 1 and Figure 2. Common to all SSGs, the average changes of velocity demands (accelerations and deceleration) and at all intensities presented were greater compared to official matches (*p* < 0.05; *d* = 2.25 − 0.71). However, in the SSGs, the players did not experience the same demands at high intensity activity (>16 km/h) and sprinting (>21 km/h), except SSG4.

## 4. Discussion

The aim of the present research was to ascertain the external (eTL) and internal (iTL) training load of different SSGs designed by a coach, to compare the demands between SSGs in relation to their objective and to analyze these demands in relation to official matches during one month in the competition period of a youth national-level soccer team. The main differences between official matches and the proposal SSGs are in relation to the analyzed variables with a moderate-to-high effect size. Besides, SSG1 (maintaining possession of the ball) and SSG4 (maintaining possession, progressing and ending in a goal with a goalkeeper) presented the highest demands in internal and external load. In contrast, SSG3 presented the lowest demands. In sport science, there are many investigations related with SSGs and official matches in elite-level soccer players. However, a lack of research in youth soccer players has been found, particularly using inertial measurement units to record the data.

### 4.1. SSGs Load Profile

In relation to the first objective, in the analyzed SSGs efforts predominate where the HR_MAX_ is between 80–90%, where movements are performed at low intensity (<14 km/h) and the neuromuscular load is obtained by low impacts (5–6 G). In the internal load analysis, many investigations found moderate-to-high intensity heart rates (80–88% HR_MAX_) in 6 vs. 6 situations [8,20,50], these values being similar to those obtained in this study. Referring to external load analysis, in time-motion variables, different authors have shown that the intensity of displacements is low (<16 km/h) due to the reduced game area, with the meters traveled at high intensity representing 15–20% of the total performed and sprinting (>21 km/h) a residual value lower than 5% [5,20,29]. Finally, the neuromuscular load analyzed through the impacts is similar to the results obtained by Abade et al. (2014) in training using SSGs in U-15, U-17 and U-19 soccer teams. Therefore, thanks to the results obtained in the present study and the reviewed research in the sport science area, it is possible to establish a specific demands profile of SSGs in situations of equality (6 vs. 6), in a small-sided pitch commonly used by soccer professionals (≈800 m^2^), and where the players’ area is reduced in relation to official games (≈65 m^2^ vs. ≈300 m^2^).

### 4.2. Comparative Analysis between SSG Objectives

Then, in the comparison among the SSGs, SSG1 and SSG4 presented the highest demands. In these tasks, the greater demands were found at high intensity (>16 km/h) and sprinting (>21 km/h), in high-to-very high impacts (>8 G) and in high-intensity heart rate zones (>80% HR_MAX_). SSG3 presented the lowest demands, with requirements in the low intensity zone in all variables. In the comparison between progressing (SSG2) and ending in two mini-goals (SSG3), Halouani et al. (2017) found that SSG2 presented higher demands than SSG3, this data being similar to the present research. This behavior is due to the need to defend a smaller area in SSG3 (zone of mini-goals vs. entire goal line). In the tasks with or without a goalkeeper (SSG4 vs. SSG3), the presence of a goalkeeper and ending in an official goal provoked a higher load, a result that coincides with previous research [1,5], but other investigations found opposite results [17,19]. The disparity of results in this comparison (SSG3 vs. SSG4) is due to the motivation aroused by the presence/absence of the goalkeeper in the game (Dellal et al., 2011). Finally, regarding the comparison between SSG1 (maintaining possession) with respect to the rest of the proposed SSGs, no research has been found that analyzes this aspect. Since with the objective of maintaining possession of the ball there are no specific roles or clear goals for progression, less organization and an incorrect distribution was evident in relation to official games, aspects that are related to greater demands, desynchronization, and worse results [51,52]. Therefore, the objective of the SSGs has an enormous influence on internal and external load demands and should be considered for the correct planning of training sessions, the tactical complex of tasks not being a direct influence on their demands.

### 4.3. Comparative Analysis among SSGs and Official Matches

In relation to SSGs versus official matches, there are significant differences among SSG1, SSG2 and SSG3 and competition demands. Specifically, SSG2 and SSG3 presented lower demands, while SSG1 produced higher demands than competition. Although, the SSGs were designed to reproduce the specific demands of a soccer game [50], the results show that there is overtraining of the changes of speed, as a consequence of the reduced dimensions of the game area [8,10,33]. Moreover, exceeding the training load can cause injuries [7]. Finally, the SSG4-vs-match comparison in internal load recorded using HR produced similar demands to official games, representing an efficient stimulus for training the aerobic adaptations required in soccer [33]. Therefore, the recommendation is to design the specific objective of training tasks [18] as they influence demands, should be motivating for players [42], and have external feedback [8] as well as producing significant adaptations in a real game context, maintaining distribution and avoiding small areas and reduced numbers of players [3,33].

While the results of this study have provided information regarding the influence of the objective in the internal and external load demands during small-sided games and their relationship with competition load, some limitations to the study must be acknowledged. One of the limitations in this study concerns the sample studied; it would be interesting to extend this study to include more participants, different categories and levels. It would also be interesting for futures studies to analyze the influence of coach feedback, pitch dimensions and orientation, number of players per team and the presence of wildcard players on the small-sided games with the objectives proposed in this research

## 5. Conclusions

From the results obtained, we can propose three considerations about the use of small-sided games (SSG) during training sessions:

- SSG are a training methodology where low-intensity movements (<14 km/h), low-intensity impacts (<6 G) and medium-high intensity heart rate demands (80–90% HR_MAX_) are predominant.
- These game-based tasks produced overtraining in changes of speed and reduced demands in high intensity movements compared to official matches.
- The objective of the SSGs directly influenced the internal and external load demands of the soccer players.

Finally, it is recommended to monitor SSGs during training sessions to obtain objective data about the specific load of each task. From these data, the correct application of SSGs in relation to the objective during the training process will achieve performance enhancement both in the soccer players and team behavior. Therefore, more research in this area is necessary for a better understanding of their effects on technical, tactical, psychological and physiological aspects.

## Figures and Tables

**Table 1:** Independent variables recorded in the investigation.

Activity	Variable	Objective	Situation	Figure
Small-sided games (25 × 40 m)	SSG1	Maintain possession of the ball	6 vs. 6	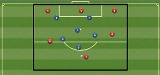
	SSG2	Maintain possession of the ball and progress to the goal line of the opposing team.	6 vs. 6	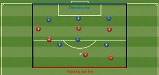
	SSG3	Maintain possession, progress to the goal-line of the opponent and try to score a goal in two 2-m mini-goals located in the pitch corners.	6 vs. 6	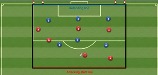
	SSG4	Maintain possession, progress to the goal line of the opponent and try to score a goal in an official goal defended by a goalkeeper (gk)	(6 + gk) vs. 6	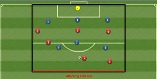
Official matches	Total match	Total demands of the match (Playing system: 4-2-3-1)	11 vs. 11	

**Table 2:** Dependent external and internal load variables recorded in the investigation.

Load	Type	Variable	Sub-Variable	Acronym	Description
External load (eTL)	Kinematical	Relative distance (m/min)	Total	m/min	Total distance covered per minute
			Walking	[0.7–7 km/h] m/min	Total distance covered between 0–7 km/h per minute
			Jogging	[7–14 km/h] m/min	Total distance covered between 7–14 km/h per minute
			Running	[14–21 km/h] m/min	Total distance covered between 14–21 km/h per minute
			Sprinting	[>21 km/h] m/min	Total distance covered between >21 km/h per minute
		HIA (%)		%HIA	Percentage of total distance traveled up to 16 km/h
		Speed (km/h)		Speed_AVG_	Average speed
		Accelerations (n/min)	Total	Acc/min	Total positive speed changes per minute
			Low	[1–2.5 m/s^2^] n/min	Total positive speed changes between 1–2.5 m/s^2^ per minute
			Medium	[2.5–4 m/s^2^] n/min	Total positive speed changes between 2.5–4 m/s^2^ per minute
			High	[>4 m/s^2^] n/min	Total positive speed changes between >4 m/s^2^ per minute
		Decelerations (n/min)	Total	Dec/min	Total negative speed changes per minute
			Low	−[1–2.5 m/s^2^] n/min	Total negative speed changes between 1–2.5 m/s^2^ per minute
			Medium	−[2.5–4 m/s^2^] n/min	Total negative speed changes between 2.5–4 m/s^2^ per minute
			High	−[>4 m/s^2^] n/min	Total negative speed changes between >4 m/s^2^ per minute
		Metabolic power (W/kg/min)		MP/min	Energy expended by the player per minute per kg
	Neuromuscular	PlayerLoad (a.u./min)		PL/min	Vector sum of device accelerations in the 3-axes per minute
		Impacts (Imp/min)	Total	Imp/min	Total impacts recorded per minute
			Very low	[5–6 G] Imp/min	Total impacts recorded between 5–6 G per minute
			Low	[6–6.5 G] Imp/min	Total impacts recorded between 6–6.5 G per minute
			Medium	[6.5–7 G] Imp/min	Total impacts recorded between 6.5–7 G per minute
			High	[7–8 G] Imp/min	Total impacts recorded between 7–8 G per minute
			Very high	[8–10 G] Imp/min	Total impacts recorded between 8–10 G per minute
			Severe	[>10 G] Imp/min	Total impacts recorded >10 G per minute
Internal load (iTL)		Heart rate (bpm)	Total (bpm)	HR_AVG_	Total average beats-per-minute
			Very low	[50–60%] HR_MAX_	Percentage of heart rate between 50–60% in relation to HR_MAX_
			Low	[60–70%] HR_MAX_	Percentage of heart rate between 60–70% in relation to HR_MAX_
			Medium	[70–80%] HR_MAX_	Percentage of heart rate between 70–80% in relation to HR_MAX_
			High	[80–90%] HR_MAX_	Percentage of heart rate between 80–90% in relation to HR_MAX_
			Very high	[90–95%] HR_MAX_	Percentage of heart rate between 90–95% in relation to HR_MAX_
			Maximum	[95–100%] HR_MAX_	Percentage of heart rate between 95–100% in relation to HR_MAX_

**Table 3:** Results of exploratory analysis of dependent variables in the present research.

Variables	*Kolmogorov-Smirnov*					*Levene*	
	SSG1	SSG2	SSG3	SSG4	OM	*F*	*p*
Relative distance (m/min)	0.12	0.12	0.01 *	0.20	0.02 *	24.13	0.00 ^†^
Walking (m/min)	0.05 *	0.17	0.20	0.20	0.20	1.82	0.12
Jogging (m/min)	0.08	0.20	0.00 *	0.12	0.20	8.09	0.00 ^†^
Running (m/min)	0.20	0.06	0.00 *	0.20	0.06	7.48	0.00 ^†^
Sprinting (m/min)	0.00 *	0.00 *	0.00 *	0.00 *	0.00 *	16.41	0.00 ^†^
HIA (%)	0.01 *	0.03 *	0.00 *	0.20	0.14	6.58	0.00 ^†^
Speed_AVG_ (km/h)	0.02 *	0.20	0.00 *	0.20	0.00 *	24.02	0.00 ^†^
Relative accelerations (n/min)	0.02 *	0.06	0.03 *	0.20	0.20	2.68	0.03 ^†^
Low acc (n/min)	0.09	0.09	0.07	0.20	0.20	3.02	0.01 ^†^
Medium acc (n/min)	0.16	0.02 *	0.10	0.20	0.00 *	2.59	0.03 ^†^
High acc (n/min)	0.03 *	0.00 *	0.00 *	0.00 *	0.00 *	1.28	0.27
Relative decelerations (n/min)	0.03 *	0.20	0.03 *	0.20	0.20	5.35	0.00 ^†^
Low dec (n/min)	0.16	0.03 *	0.00 *	0.20	0.20	3.84	0.00 ^†^
Medium dec (n/min)	0.01 *	0.04 *	0.20	0.00 *	0.00 *	2.91	0.02 ^†^
High dec (n/min)	0.07	0.00 *	0.00 *	0.07	0.00 *	5.60	0.00 ^†^
Metabolic Power (W/kg/min)	0.20	0.20	0.02 *	0.15	0.00 *	26.29	0.00 ^†^
PlayerLoad (PL/min)	0.20	0.20	0.00 *	0.05 *	0.00 *	11.73	0.00 ^†^
Relative impacts (n/min)	0.00 *	0.00 *	0.00 *	0.02 *	0.05 *	0.51	0.72
Very low imp (n/min)	0.05 *	0.02 *	0.00 *	0.20	0.20	0.93	0.44
Low imp (n/min)	0.00 *	0.00 *	0.00 *	0.00 *	0.00 *	1.80	0.13
Medium imp (n/min)	0.00 *	0.00 *	0.00 *	0.00 *	0.00 *	1.44	0.22
High imp (n/min)	0.00 *	0.00 *	0.00 *	0.00 *	0.00 *	1.13	0.34
Very high imp (n/min)	0.00 *	0.00 *	0.00 *	0.00 *	0.00 *	1.77	0.13
Severe imp (n/min)	0.00 *	0.00 *	0.00 *	0.00 *	0.11	1.06	0.38
HR_AVG_ (bpm)	0.09	0.20	0.20	0.20	0.20	4.35	0.00 ^†^
Very low (%)	0.00 *	0.00 *	0.00 *	0.00 *	0.00 *	9.48	0.00 ^†^
Low (%)	0.00 *	0.00 *	0.00 *	0.00 *	0.00 *	14.79	0.00 ^†^
Medium (%)	0.00 *	0.00 *	0.00 *	0.00 *	0.20	5.57	0.00 ^†^
High (%)	0.00 *	0.11	0.00 *	0.20	0.20	2.87	0.02 ^†^
Very high (%)	0.00 *	0.00 *	0.00 *	0.00 *	0.20	4.70	0.00 ^†^
Severe (%)	0.00 *	0.00 *	0.00 *	0.00 *	0.00 *	0.56	0.68

Note. SSG: Small-sided game; OM: Official match; HIA: High-intensity activity; Acc: Accelerations; Dec: Decelerations; Imp: Impacts; HR: Heart rate; *F*: F-value of Levene test; *p*: p-value. * *p* < 0.05 reject null hypothesis. The sample is non-normal. ^†^ *p* < 0.05 reject null hypothesis. There is no homogeneity of variables.

**Table 4:** Descriptive and comparative analysis of small-sided games and official matches in the external and internal load variables registered in the investigation.

Variable	SSG1	SSG2	SSG3	SSG4	Official Match	Diff	ES
	M ± SD	M ± SD	M ± SD	M ± SD	M ± SD		
Distance (m/min)	127.38 ± 15.35	109.26 ± 21.29	94.08 ± 34.69	117.52 ± 20.04	98.30 ± 21.94	A, C, F	1.24–0.53
Walking (m/min)	48.50 ± 6.89	53.77 ± 7.61	48.05 ± 9.24	51.71 ± 8.27	45.67 ± 6.80	A, D	0.73–0.42
Jogging (m/min)	58.44 ± 14.94	43.74 ± 18.22	34.55 ± 23.29	47.75 ± 15.25	36.36 ± 12.49	A, B, C, F	1.22–0.44
Running (m/min)	19.36 ± 7.61	11.27 ± 7.50	10.96 ± 11.00	16.04 ± 7.30	13.62 ± 5.70	A, C, E, F	1.07–0.45
Sprinting (m/min)	1.07 ± 1.51	0.48 ± 1.03	0.52 ± 1.19	2.03 ± 2.89	2.65 ± 1.95	A, B, E, F	0.71–0.40
HIA (%)	8.26 ± 4.32	5.12 ± 3.88	5.43 ± 5.68	8.67 ± 4.65	9.74 ± 4.21	A, B, E, F	0.83–0.56
Speed_AVG_ (km/h)	6.75 ± 0.68	5.86 ± 1.05	5.19 ± 1.57	6.26 ± 0.85	5.95 ± 0.94	A, B, C, F	1.29–0.42
Accelerations (n/min)	18.47 ± 3.88	15.60 ± 3.70	14.94 ± 4.97	16.20 ± 3.73	12.42 ± 2.28	A, B, C	0.79–0.60
Low acc (n/min)	14.39 ± 3.39	12.40 ± 3.24	12.12 ± 4.41	12.53 ± 3.18	10.45 ± 2.03	A, B	0.60–0.57
Medium acc (n/min)	3.45 ± 0.97	2.69 ± 0.87	2.40 ± 0.92	3.15 ± 0.86	1.65 ± 0.47	A, B, F	1.11–0.53
High acc (n/min)	0.63 ± 0.38	0.51 ± 0.30	0.41 ± 0.35	0.52 ± 0.36	0.32 ± 0.14	B	0.60
Decelerations (n/min)	16.22 ± 3.63	13.40 ± 3.33	12.44 ± 5.05	14.15 ± 3.49	10.85 ± 2.26	A, B	0.86–0.58
Low dec (n/min)	13.00 ± 3.41	10.72 ± 2.93	10.31 ± 4.38	11.30 ± 3.19	9.14 ± 1.95	A, B	0.72–0.51
Medium dec (n/min)	2.55 ± 0.93	2.18 ± 0.90	1.76 ± 0.98	2.26 ± 0.69	1.28 ± 0.43	A, B	0.83–0.40
High dec (n/min)	0.67 ± 0.36	0.50 ± 0.35	0.37 ± 0.38	0.60 ± 0.33	0.44 ± 0.18	B, F	0.81–0.48
Metabolic Power (W/kg/min)	8.13 ± 0.88	6.86 ± 1.34	5.85 ± 2.12	7.44 ± 1.23	6.24 ± 1.31	A, B, F	1.40–0.45
PlayerLoad (PL/min)	1.47 ± 0.25	1.21 ± 0.33	1.06 ± 0.43	1.34 ± 0.28	1.80 ± 2.95	A, B, F	1.17–0.42
Impacts (n/min)	13.93 ± 8.32	10.71 ± 7.28	8.91 ± 8.21	13.11 ± 8.42	10.46 ± 6.62	B, F	0.61–0.41
Very low imp (n/min)	8.42 ± 4.41	6.29 ± 4.25	5.21 ± 4.49	7.39 ± 3.81	5.78 ± 3.26	A, B, F	0.72–0.49
Low imp (n/min)	2.10 ± 1.50	1.56 ± 1.13	1.33 ± 1.42	2.06 ± 1.71	1.50 ± 0.99	B, F	0.53–0.41
Medium imp (n/min)	1.37 ± 1.23	1.13 ± 0.98	0.89 ± 1.16	1.32 ± 1.13	1.04 ± 0.76	B, F	0.40–0.38
High imp (n/min)	1.31 ± 1.23	1.03 ± 0.99	0.89 ± 1.08	1.38 ± 1.28	1.15 ± 1.00	B, F	0.41–0.36
Very high imp (n/min)	0.59 ± 0.75	0.51 ± 0.65	0.47 ± 0.69	0.76 ± 1.07	0.75 ± 0.81		
Severe imp (n/min)	0.14 ± 0.23	0.18 ± 0.28	0.12 ± 0.23	0.21 ± 0.34	0.24 ± 0.20		
HR_AVG_ (bpm)	167.05 ± 12.33	161.14 ± 14.19	155.07 ± 17.71	164.88 ± 12.68	161.48 ± 15.10	A, B, F	0.79–0.44
Very low (%)	2.82 ± 5.29	2.21 ± 3.58	5.61 ± 11.78	1.88 ± 3.85	6.76 ± 9.47		
Low (%)	7.07 ± 9.96	12.58 ± 15.17	19.77 ± 19.47	8.21 ± 10.87	11.15 ± 14.34	A, F	0.82–0.41
Medium (%)	18.78 ± 19.64	28.32 ± 22.49	31.18 ± 24.79	24.83 ± 21.87	20.21 ± 10.68	A, B	0.55–0.45
High (%)	40.76 ± 23.41	37.69 ± 25.69	26.80 ± 26.26	41.73 ± 22.69	36.00 ± 18.14	B, D, F	0.61–0.42
Very high (%)	22.02 ± 21.46	11.48 ± 18.11	9.49 ± 17.12	14.77 ± 17.06	15.03 ± 10.48	A, B	0.65–0.53
Severe (%)	6.45 ± 14.88	5.82 ± 17.75	5.26 ± 14.85	6.50 ± 14.93	8.12 ± 13.97		

Diff: Pairwise comparison; ES: Effect size; SSG: Small-sided game; OM: Official match; HIA: High-intensity activity; Acc: Accelerations; Dec: Decelerations; Imp: Impacts; HR: Heart rate. A: Differences between SSG1 and SSG2 (*p* < 0.05); B: Differences between SSG1 and SSG3 (*p* < 0.05); C: Differences between SSG1 and SSG4 (*p* < 0.05); D: Differences between SSG2 and SSG3 (*p* < 0.05); E: Differences between SSG2 and SSG4 (*p* < 0.05); F: Differences between SSG3 and SSG4 (*p* < 0.05).
